# Nutrient adequacy and nutrient sources of adults among ninety-two First Nations communities across Canada

**DOI:** 10.17269/s41997-021-00490-y

**Published:** 2021-06-28

**Authors:** Malek Batal, Hing Man Chan, Amy Ing, Karen Fediuk, Peter Berti, Tonio Sadik, Louise Johnson-Down

**Affiliations:** 1 Département de nutrition, Faculté de Médecine, Université de Montréal, Pavillon Liliane de Stewart, CP 6128 succ. Centre-Ville, Montréal, QC H3T 1A8 Canada; 2 Centre de recherche en santé publique de l’Université de Montréal et du CIUSS du Centre-sud-de-l’Île-de-Montréal (CReSP), 7101 Avenue du Parc, Montréal, QC H3N 1X7 Canada; 3 Department of Biology, University of Ottawa, 30 Marie Curie, Ottawa, ON K1N 6N5 Canada; 4 First Nations Food, Nutrition and Environment Study, University of Ottawa, 30 Marie Curie, Ottawa, ON K1N 6N5 Canada; 5 HealthBridge Foundation of Canada, 1 Nicholas Street, Suite 1004, Ottawa, ON KIN 7B7 Canada; 6 Assembly of First Nations, 55 Metcalfe Street, Suite 1600, Ottawa, ON K1P 6L5 Canada

**Keywords:** Indigenous, First Nations, Diet quality, Recommended dietary allowances, Nutrients, Traditional food, Autochtones, Première Nations, apports nutritionnels de référence, nutriments, aliments traditionnels

## Abstract

**Objectives:**

To identify food sources of nutrients in First Nations adults in Canada and to establish whether these populations are meeting their nutrient requirements and whether traditional foods (TF) contribute to better nutrient intake.

**Methods:**

The First Nations Food, Nutrition and Environment Study is a cross-Canada participatory study of First Nations adults living south of the 60^th^ parallel. Twenty-four-hour recalls were conducted in 92 First Nations communities from 2008 to 2016. Repeat recalls were attempted with 20% of participants to adjust for within-person variation and estimate the proportion of individuals below recommendations according to Institute of Medicine guidelines. Nutrients from days with and without TF were compared. The main food sources of select nutrients were identified, including TF.

**Results:**

Mean energy intakes among women and men ranged from 1664 to 1864 and from 1761 to 2298 kcal/day respectively. Most macronutrients were within the acceptable macronutrient diet range except for fat in most age groups and carbohydrates in men 71 years of age and older. Saturated fat was above recommendations for all ages. Only niacin was identified as above recommendations in all age and sex categories. Days where TF were eaten showed greater intakes of key nutrients.

**Conclusion:**

It is imperative that we find culturally appropriate ways to improve the quality and nutritional value of First Nations Peoples food intake by improving TF access and use on the one hand and conversely providing better quality store-bought foods. For success in this, we must empower First Nations communities and health practitioners to collaboratively overcome these challenges.

## Résumé

**Objectifs:**

Établir si l’alimentation des adultes des Premières Nations au Canada est satisfaisante et si les aliments traditionnels (AT) contribuent à un meilleur apport en nutriments et identifier les sources alimentaires des nutriments.

**Méthode:**

L’étude sur l’alimentation, la nutrition et l’environnement chez les Premières Nations est une étude participative pancanadienne visant les adultes des Premières Nations vivant au sud du 60^e^ parallèle. Des rappels de 24 heures ont été effectués dans 92 communautés des Premières Nations de 2008 à 2016. Les rappels ont été répétés avec 20 % des participants pour ajuster la variation intra-individuelle et estimer la proportion d’individus à risque de consommation insuffisante de nutriments selon les apports nutritionnels de référence de l’« Institute of Medicine ». Les sources alimentaires (incluant les AT) de certains nutriments ont été identifiées.

**Résultats:**

La moyenne des apports énergétiques moyens chez les femmes et les hommes variaient de 1 664 à 1 864 et 1 761 à 2 298 kilocalories/jour respectivement. La plupart des macronutriments se situaient dans l’intervalle établi par l’étendue des valeurs acceptables des macronutriments, à l’exception des lipides dans la plupart des groupes d’âge et des glucides chez les hommes de 71 ans et plus. Les acides gras saturés étaient supérieurs aux recommandations pour tous les âges. Seule la niacine était supérieure au besoin moyen estimatif. Les jours où les AT sont consommés, une augmentation notable dans les apports en certains nutriments-clés est observée.

**Conclusion:**

Il est important de trouver des moyens pour améliorer la qualité et la valeur nutritionnelle de l’apport alimentaire des Peuples des Premières Nations en respectant leur culture et en offrant un meilleur accès et une utilisation améliorée des AT d’une part et en fournissant des aliments provenant du magasin de meilleure qualité d’autre part. Pour réussir, les communautés des Premières Nations et les professionnels de la santé devraient être impliqués de manière collaborative.

## Introduction

Indigenous peoples throughout the world continue to face health disparities as compared with non-Indigenous populations (Adelson 2005). These health disparities result from a long list of social, environmental and lifestyle factors (Batal et al. 2021c; Ford 2012; McNally and Martin 2017; Mikkonen and Raphael 2010). Life expectancy in First Nations is lower as they cope with a high burden of nutrition-related chronic disease (NRCD) such as obesity, insulin resistance, type 2 diabetes, and cardiovascular disease (Kolahdooz et al. 2015; McNally and Martin 2017). Overweight and obesity are more prevalent in First Nations (74%) than in the general Canadian population (61%) (First Nations Information Governance Centre 2012; Statistics Canada 2009). Prevalence of diabetes in First Nations in Canada is 19% (Batal et al. 2021c).

Traditional foods (TF) that are cultivated, fished, hunted, or gathered are culturally important and are valuable sources of nutrients to First Nations individuals in Canada despite their contaminant burden (Johnson-Down and Egeland 2013; Juric et al. 2017; Willows 2005). These foods are important to the health and wellness of First Nations Peoples as they contribute to greater physical activity, cultural connectedness, and community pride (Batal et al. 2021b; Willows 2005). Diets of First Nations have historically gone through a nutrition transition characterized by an increased intake of store-bought market foods (MF) and a decrease in TF that has contributed to the health challenges they face (Egeland and Harrison 2013). Regrettably, MF have contributed to poorer diets with higher intakes of fat, saturated fat and sodium. Previous studies in a subset of this study population showed that the contribution of energy from nutrient-poor ultra-processed products (UPP) was 54% and that TF decreased with increasing UPP (Batal et al. 2018b). UPP are often more attractive because of their low cost and ease of use (Batal et al. 2018a, b).

Food insecurity is very high in First Nations Peoples in Canada (Willows et al. 2009; Willows et al. 2019). Food sovereignty of these Peoples has been affected by colonial policies (e.g., limiting access to land and gathering of TF, and residential schools), industrial development, and climate change (Coté 2016). Some of the factors contributing to this include remoteness of communities, lack of resources for buying MF and obtaining TF due to insufficient employment, and high food costs, especially in rural and remote communities (Batal et al. 2021b).

The objectives of this article are to describe the nutrient and food intake of First Nations adults living on-reserve in Canada and to establish whether these populations are meeting their nutrient requirements as compared with Institute of Medicine guidelines and whether TF are contributing to better nutrient intake.

## Methods

On-reserve First Nations adults living below the 60^th^ parallel across Canada were sampled and recruited for the participatory First Nations Food, Nutrition and Environment Study (FNFNES) (Batal et al. 2018a, b; Chan et al. 2021). Methodology workshops were held in each region with community representatives and research tools were accordingly built and adapted in response to community feedback when needed. In addition, study objectives and methods were presented to the leadership and members of each participating First Nation and a Band Council Resolution was passed before data collection could take place (Chan et al. 2021). Multi-stage sampling was conducted (based on ecozones, communities and households) and is described elsewhere (Chan et al. 2021). First Nations principles of Ownership, Control, Access and Possession (OCAP®) were followed (Chan et al. 2021; Schnarch 2004). Informed consent was obtained from all individuals (Chan et al. 2021). Individual community results were reported back to each community by the study’s principal investigators and discussed with community members and leadership to ascertain they were relevant (Chan et al. 2021). Community results remained confidential and regional results were anonymized, grouped and then presented at regional events to First Nations representatives to incorporate their feedback before any publication.

Interviews were conducted in 92 communities between the years 2008 and 2016 by trained First Nations community workers under the guidance of a trained dietitian and included information on diet, lifestyle, environmental concerns, health, and food security. Height and weight were both measured and self-reported. Twenty-four-hour recalls were conducted using a 3-stage multiple pass method: a quick list of foods and beverages, followed by a more detailed description, including the amounts eaten, and then a final review (Raper et al. 2004). Portion sizes were estimated using 3-dimensional food models based on models developed by Santé Québec (Santé Québec, Montreal, QC). Alcohol was excluded from all dietary intake analyses. A second 24-h recall was attempted for a 20% subsample of the population and completed in 1247 of 6201 (20.1%) individuals: repeat recalls were conducted on a different day of the week with a range of 1 to 104 days between recalls and a mean interval of 32±20 days.

Data from interviews were entered into a study database using Epi Info 3.5.4 (Centers for Disease Control and Prevention, Atlanta, GA, USA, 1988). The 24-h recall data were entered by research nutritionists at the Université de Montréal, using CANDAT (Godin, London, ON) and the 2010 Canadian Nutrient File (CNF) (Health Canada 2010). A database of nutrient data not available from the CNF was compiled for missing TF and MF; data for TF were added from published literature or imputed from similar foods whereas MF were researched using food labels. The data entry for the recalls was cross-checked line by line against the transcribed recalls in 10% of each community’s recalls, and discrepancies were corrected: if many errors were found during this initial cross-check, a further 10% check was conducted. A range error check was also performed to check for outliers in grams of food intake, unusual foods, energy and nutrient intakes.

A total of 6487 interviews were completed with the nutrient data from 286 individuals excluded from the analyses (245 pregnant and/or lactating women, 27 participants with missing age and age group values and 14 participants with zero kcal intake) thereby leaving data for 6201 individuals with dietary data from 24-h recalls.

Nutrient adequacy and quality were obtained by comparing the 24-h recall to the Dietary Reference Intakes (DRIs) (Institute of Medicine 2000a, 2001, 2005a, b, 2011; National Academies of Sciences Engineering and Medicine 2019; U.S. Department of Health and Human Services and U.S. Department of Agriculture 2015). Because our goal was to compare with the Canadian Community Health Survey, the SIDE (Software for Intake Distribution Estimation) SAS subroutine (Iowa State University, Ames, Iowa, 2001) was performed on 6201 participants (4010 women and 2191 men) to obtain the distributions of usual intake by sex in three age groups: 19–50, 51–70, and 71 years and over (Guenther et al. 1997; Institute of Medicine 2000b; Statistics Canada 2006). The SIDE approximates usual intake by accounting for within-individual random error, and it was used to assess nutrient adequacy. The 95th percent confidence intervals (CI) for the proportion of participants with intakes either below the Estimated Average Requirements (EAR), above the Tolerable Upper Intake Level (UL) or within the Accepted Macronutrient Distribution Range (AMDR) were obtained by ordering the 500 bootstraps iterations in order of percent below the EAR (or percent above the UL, or percent within the AMDR) and using the 12^th^ lowest iteration (i.e., the lowest 2.5%) as the lower end estimate and the 12^th^ highest iteration (i.e., the highest 2.5%) as the upper end estimate. For some sex and age groups, the estimate of the percentile value, as well as the level of adequacy, could not be estimated precisely enough due to the high level of variability in nutrient intake between and within individuals. When the coefficients of variance (CV) for the percent below EAR were greater than 33%, the values were suppressed.

Food groupings (including TF) were established using their main food component (e.g., chicken, pork, beef, fish, shellfish, milk, cheese, yogourt, fruit, vegetable, legumes, cereal, carbonated drinks, bread, chocolate bars, pasta) to obtain the top food sources of nutrients, resulting in 67 groups. The proportion of each nutrient was calculated and then the food groups were ranked. Only the food groups that ranked in the top 10 for the nutrients were reported. The proportion of individuals taking nutritional supplements was obtained from a question on the Socio/Health/Lifestyle questionnaire. Estimation of TF intake included land animals (e.g., moose, caribou, bear and beaver), wild birds (e.g., geese, ptarmigan), fish and shellfish (e.g., salmon, trout, clams), berries (e.g., blueberries, raspberries), cultivated crops (e.g., the three sisters, i.e., companion crops cultivated in First Nations communities in southern Quebec and southern Ontario (corn, beans and squash)) and other foods as identified by the community members.

Data analysis used SAS/STAT version 9.4 (SAS, Cary, NC, USA, 2013). All analyses were weighted by community, household and individual for non-response and to ensure they represented this population (Chan et al. 2021). Data were also adjusted for changes in population from 2008 to 2017 using a ratio of the 2017 population and the reference year (Chan et al. 2021).

## Results

Average energy intakes ranged from 1664 to 1864 kcal/day and from 1761 to 2298 kcal/day among women and men respectively (Table 1). The mean percentage of energy from protein was within the AMDR for both sexes and all age groups (16.6% to 22.4%) (Table 1) (Institute of Medicine 2005a). The mean percentage of energy from carbohydrates was within the recommended range for women and for men aged 19–50 and 51–70 years (Table 1); however, men aged 71 years and over had a mean intake of carbohydrates below the AMDR (Institute of Medicine 2005a). The mean intake of percentage energy from fat was above the recommended range for five of the six age-sex groups; women aged 51–70 years were within recommendations (Institute of Medicine 2005a). The mean and median intakes of percentage of energy from saturated fat were above the recommended 10% for all age and sex groups (World Health Organization 2018).

**Table 1:** Nutrient intakes and percent within acceptable macronutrient diet range (AMDR) of Canadian First Nations participants from the First Nations Food, Nutrition and Environment Study (FNFNES) 2008–2018

Nutrient	Men							Women						
	AMDR	19–50 years		51–70 years		71 years and over		AMDR	19–50 years		51–70 years		71 years and over	
		Mean (SE)	% within AMDR [95% CI]	Mean (SE)	% within AMDR [95% CI]	Mean (SE)	% within AMDR [95% CI]		Mean (SE)	% within AMDR [95% CI]	Mean (SE)	% within AMDR [95% CI]	Mean (SE)	% within AMDR [95% CI]
Energy (kcal)	NA	2298 (53)	NA	1948 (70)	NA	1761 (146)	NA	NA	1864 (39)	NA	1669 (61)	NA	1664 (81)	NA
Percent energy as protein (%)	10−35	18.3 (0.7)	99.7 [98.2−100]	20.6 (1.3)	99.4 [95.2−100]	22.4 (1.5)	NE	10−35	16.6 (0.2)	NE	18.4 (0.4)	100 [99.3−100]	18.1 (0.8)	100 [98.1−100]
Percent energy as carbohydrate (%)	45−65	48.5 (0.8)	70.1 [63.4−88.8]	47.9 (1.4)	52.8 [44.9−71.1]	43.3 (1.8)	NE	45−65	48.9 (0.5)	NE	48.0 (0.6)	72.9 [63.3−85.9]	47.5 (1.4)	77.2 [49.8−100]
Percent energy as fat (%)	20−35	36.0 (1.0)	52.1 [29−64.3]	38.3 (3.2)	NE	35.3 (1.5)	NE	20−35	35.7 (0.3)	NE	34.6 (0.4)	NE	35.2 (1.1)	NE
Percent energy as saturated fat (%)	NA	11.3 (0.2)	NA	11.1 (0.3)	NA	11.2 (0.7)	NA	NA	11.4 (0.1)	NA	10.9 (0.2)	NA	10.6 (0.3)	NA

*NA*, not applicable; *NE*, not estimable using SIDE software

First Nations adults appeared to have intakes for niacin that were above the EAR (Table 2). The main sources of niacin were game meat (any wild game), chicken, white bread, beef, pork, pasta, fish, cold cuts/sausages, pizza, and mixed dishes (data not shown). Meeting the dietary requirements could not be determined due to a high CV for carbohydrates, iron, vitamin B12, thiamin, riboflavin, and phosphorus. Intakes were below recommendations for vitamins A, D, and C, and for folate, calcium, and magnesium (Tables 1 and 2) (Institute of Medicine 1997, 1998, 2000a, 2001, 2005a, 2011). There were intakes below recommendations for vitamin B6 among women and men aged 51–70 years (Table 2) (Institute of Medicine 1998). Among the four nutrients with a recommended average daily intake level (AI), intakes were below the AI for fibre, potassium, and linoleic acid (Table 2) (Institute of Medicine 2005a, b). Women and men aged 19–70 years had mean intakes greater than the AI for linolenic acid, suggesting adequate intake (Institute of Medicine 2005a). Intakes did not exceed ULs for any of the seven nutrients with ULs (Institute of Medicine 1998, 2000a, 2001, 2011). Although sodium no longer has a UL, age-sex groups ranged from 51.1% to 95.5% above the Chronic Diseases Risk Reduction intake of 2300 mg (National Academies of Sciences Engineering and Medicine 2019).

**Table 2:** Nutrient intakes and percent below estimated average requirements (EAR) or percent above adequate intakes (AI) of Canadian First Nations participants from the FNFNES, 2008−2018^a^

Nutrient	Men							Women						
	EAR/AI	19−50 years		51−70 years		71 years and over		EAR/AI	19−50 years		51−70 years		71 years and over	
		Mean (SE)	% below EAR or above AI [95% CI]	Mean (SE)	% below EAR or above AI [95% CI]	Mean (SE)	% below EAR or above AI [95% CI]		Mean (SE)	% below EAR or above AI [95% CI]	Mean (SE)	% below EAR or above AI [95% CI]	Mean (SE)	% below EAR or above AI [95% CI]
Carbohydrates^b^ (g)	100	274 (7)	[-*]	226 (12)	[-*]	188 (12)	[-*]	100	225 (5)	[-*]	197 (7)	[-*]	194 (10)	[-*]
Fibre^c^ (g)	30−38	14.6 (0.4)	0 [0−0]	14.4 (0.9)	[-*]	13.3 (1.5)	[-*]	21−25	12.4 (0.3)	[-*]	12.5 (0.5)	[-*]	13.2 (0.7)	[-*]
Linoleic acid^c^ (g)	14−17	14.4 (0.5)	25.0 [14.8−32.9]	11.6 (0.6)	26.3 [15.5−33.4]	11.2 (1.5)	[-*]	11−12	12.1 (0.4)	49.6 [34.1−64.1]	10.5 (0.4)	37.6 [21.2−53.4]	11.4 (1.2)	61.6 [7.6−93.2]
Linolenic acid^c^ (g)	1.6	1.6 (0.1)	41.7 [33.7−49.0]	1.6 (0.1)	39.8 [29.4−47.5]	1.5 (0.2)	[-*]	1.1	1.4 (0.1)	**78.4 [63.1−99.7]**	1.4 (0.1)	**76.6 [59.4−98.3]**	1.4 (0.1)	81.5 [43.6−99.9]
Vitamin A^b^ (RAE)	625	491 (25)	**84.4 [75.0−99.8]**	515 (31)	**65.2 [55.9−85.5]**	537 (61)	71.8 [45.6−100]	500	430 (16)	**71.2 [64.1−85.8]**	511 (38)	60.8 [45.0−70.4]	579 (144)	54.5 [6.3−87.2]
Vitamin C^b^ (mg)	75	92 (6)	47.0 [21.6−56.7]	78 (13)	60.0 [38.9−76.3]	41 (6)	**93.7 [77.5−100]**	60	79 (4)	35.6 [24.5−46.9]	69 (7)	47.1 [27.3−62.2]	59 (12)	59.0 [27.8−92.7]
Vitamin D^b^ (μg)	10−15	4.3 (0.3)	**98.7 [94.4−100]**	5.1 (0.5)	**98.8 [88.9−100]**	6.5 (0.9)	**98.1 [87.8−100]**	10−15	3.7 (0.3)	**99.8 [98.5−100]**	3.6 (0.3)	**99.2 [96.5−100]**	5.9 (0.9)	**95.8 [91.1−100]**
Folate^b^ (DFE)	320	400 (13)	21.2 [1.3−32.3]	372 (19)	33.9 [18.8−48.9]	348 (34)	47.5 [6.2−85.8]	320	336 (11)	44.0 [30.7−57]	324 (20)	50.8 [27.7−68.3]	335 (26)	[-*]
Vitamin B6^2^ (mg)	1.1−1.4	1.8 (0.1)	[-*]	1.5 (0.1)	49.7 [31.7−62]	1.6 (0.2)	[-*]	1.1−1.3	1.4 (0.1)	21.2 [12.1−28.2]	1.3 (0.1)	52.4 [27.3−70.7]	1.4 (0.1)	47.0 [12.0−67.3]
Vitamin B12^b^ (μg)	2.0	8.1 (0.8)	0 [0−0.4]	7.0 (1.4)	[-*]	7.3 (1.4)	0 [0−1.3]	2.0	4.5 (0.2)	0 [0−1.9]	5.7 (1.4)	[-*]	4.7 (0.6)	[-*]
Thiamin^b^ (mg)	1.0	1.9 (0.1)	[-*]	1.8 (0.1)	8.8 [3.9−13.1]	1.6 (0.1)	[-*]	0.9	1.5 (0.04)	[-*]	1.5 (0.1)	[-*]	1.6 (0.1)	[-*]
Riboflavin^b^ (mg)	1.1	2.3 (0.1)	0 [0−0.5]	2.1 (0.1)	[-*]	2.0 (0.1)	[-*]	0.9	1.8 (0.04)	[-*]	1.8 (0.1)	[-*]	1.8 (0.1)	[-*]
Niacin^b^ (NE)	12	45.9 (1.2)	0 [0−0]	39.3 (1.7)	0 [0−0.3]	43.6 (5.6)	0 [0−0]	11	35 (0.7)	0 [0−0]	34.1 (1.8)	0 [0−0]	35.1 (2.4)	0 [0−0]
Calcium^b^ (mg)	800	707 (26)	**73.2 [59.0−97.1]**	618 (31)	**76.5 [69.9−84.1]**	643 (61)	**80.8 [61.9−99.7]**	800−1000	576 (16)	**93.0 [87.8−99]**	540 (15)	**99.0 [96.9−100]**	536 (45)	**97.8 [94.9−100]**
Iron^b^ (mg)	6.0	17.3 (0.6)	0 [0−0.1]	15.7 (0.6)	[-*]	15.5 (1.0)	[-*]	5.0−8.1	13.2 (0.4)	[-*]	12.7 (0.5)	0 [0−0.4]	13.2 (0.9)	0 [0−0]
Potassium^c^ (mg)	3400	2790 (74)	17.3 [8.4−25.6]	2550 (76)	12.7 [4−16.3]	2415 (160)	[-*]	2600	2236 (47)	22.3 [13.9−28.8]	2196 (107)	19.4 [8.6−36.2]	2156 (119)	19.4 [0.1−29.2]
Sodium^c^ (mg)	1500	3719 (114)	99.9 [98.9−100]	3023 (108)	96.2 [91−99.7]	2925 (175)	93.9 [85.4−100]	1500	2997 (75)	99.8 [98.9−100]	2620 (92)	96.8 [92.4−99.9]	2475 (129)	94.7 [82.6−100]
Magnesium^b^ (mg)	330−350	272 (12)^d^ 271 (7) ^e^	**87.0 [67.9−100]**^d^ **88.3 [81.0−96.6]**^e^	256 (8)	**86.9 [82.8−92.8]**	236 (12)	**99.6 [94.3−100]**	255−265	231 (8)^d^ 224 (8) ^e^	**69.7 [55.0−96.9]**^d^ **80.0 [73.2−86.4]**^e^	228 (10)	**76.1 [64.4−89.4]**	236 (12)	**82.4 [70.3−99.8]**
Phosphorus^b^ (mg)	580	1345 (37)	0 [0−0.1]	1226 (46)	[-*]	1323 (129)	0 [0−0.3]	580	1080 (21)	0 [0−0.4]	1049 (61)	[-*]	1049 (61)	[-*]
Zinc^b^ (mg)	9.4	14.2 (0.6)	[-*]	12.5 (0.5)	28.5 [9.1−39.0]	12.0 (1.4)	[-*]	6.8	10.5 (0.3)	[-*]	10.3 (0.6)	[-*]	9.8 (0.7)	[-*]

^a^All data <50% below the EAR is identified in bold and [-*] data are suppressed due to extreme sampling variability. ^b^Nutrients with an EAR. ^c^Nutrients with an AI. ^d^Mean and percent below EAR for individuals 19−30 years of age. ^e^Mean and percent below EAR for individuals 31−50 years of age

Approximately 18% of individuals reported at least one TF on the recalls. Comparisons of days with and without TF in both men and women in all age groups showed that days with TF had more protein but less fat, total and saturated, and carbohydrates (Table 3). The days with TF recalls had more iron, zinc, magnesium, potassium, phosphorus, vitamins A, B6, B12, C and D, riboflavin, and niacin but less sodium (Table 3).

**Table 3:** Comparison of days with and without traditional foods in Canadian First Nations participants from the FNFNES, 2008−2018

Nutrient	Days with TF (*n *= 1206 recalls)	Days without TF (*n *= 4995 recalls)
	Mean ± SE	Mean ± SE
Energy (kilocalories)	1964 ± 27.4	1911 ± 13.6
Protein*** (g)	117 ± 2.16	75.1 ± 0.62
Fat*** (g)	71.3 ± 1.32	78.7 ± 0.71
Carbohydrates** (g)	219 ± 3.54	231 ± 1.80
Total sugars*** (g)	70.2 ± 1.92	78.5 ± 0.93
Fibre (g)	13.0 ± 0.25	13.2 ± 0.13
Cholesterol*** (g)	382 ± 8.59	314 ± 3.83
Saturated fat*** (g)	20.5 ± 0.40	25.4 ± 0.24
Monounsaturated fat** (g)	27.7 ± 0.59	30.2 ± 0.29
Polyunsaturated fat (g)	15.2 ± 0.36	15.7 ± 0.18
Linoleic acid* (g)	11.6 ± 0.30	12.3 ± 0.15
Linolenic acid*** (g)	1.84 ± 0.06	1.38 ± 0.02
Calcium** (mg)	569 ± 10.8	609 ± 6.40
Iron*** (mg)	20.1 ± 0.41	12.9 ± 0.11
Zinc*** (mg)	17.0 ± 0.40	10.2 ± 0.10
Magnesium*** (mg)	278 ± 4.38	231 ± 1.79
Copper*** (mg)	1.63 ± 0.03	1.13 ± 0.02
Potassium*** (mg)	2908 ± 43.2	2260 ± 17.6
Sodium*** (mg)	2744 ± 55.1	3140 ± 27.3
Phosphorus*** (mg)	1489 ± 23.8	1075 ± 8.51
Vitamin A** (μg)	562 ± 32.3	454 ± 7.03
Vitamin D*** (μg)	7.62 ± 0.41	3.23 ± 0.05
Vitamin C* (mg)	88.0 ± 4.33	76.6 ± 1.72
Folate (μg)	362 ± 7.15	349 ± 3.57
Thiamin (mg)	1.63 ± 0.03	1.63 ± 0.02
Riboflavin*** (mg)	2.22 ± 0.04	1.88 ± 0.02
Niacin*** (mg)	47.7 ± 0.83	35.6 ± 0.29
Vitamin B6*** (mg)	1.72 ± 0.03	1.41 ± 0.02
Vitamin B12*** (μg)	14.0 ± 0.60	3.96 ± 0.13

*p < 0.05; **p < 0.01; ***p < 0.0001 significance in unpaired *t*-test

The most common beverages found on the recalls were coffee (422 g/day), water (tap (376 g/day) and bottled (189 g/day)) and carbonated drinks (201 g/day) while the most common foods were soup (101 g/day), pasta/noodles (62 g/day), vegetables (60 g/day), white bread/buns (54 g/day) and potatoes (48 g/day). The top foods contributing to macro- and micronutrients are outlined in Tables 4 and 5. Beef, chicken and cold cuts and sausages were among the top contributors of energy, protein, fat, and saturated fat (Table 4). Eggs were among the top contributors to vitamins A and D, folate, calcium, iron, and zinc (Table 5). Pasta/noodles were among the top contributors to vitamin D, folate, calcium, iron, sodium, and zinc (Table 5). White bread/buns were among the top contributors to folate, calcium, iron, sodium, and zinc (Table 5). Game meat was among the top contributors to iron and zinc (Table 5). About half of the iron in the diet came from white bread, cereal, wild meat, beef, and pasta (Table 5). One quarter of the vitamin D came from fish, while approximately 48% came from milk, margarine, and eggs (Table 5). Processed meats such as cold cuts and sausages were the top contributor to both total fat and saturated fat (Table 4). The main sources of salt were processed foods such as canned and dehydrated soups, white bread, and processed meats (Table 5).

**Table 4:** Top contributors to macronutrients by percent of total and ranking for Canadian First Nations participants from the FNFNES, 2008−2018

Food	Energy Percent (rank)	Protein Percent (rank)	Fat Percent (rank)	Carbohydrates Percent (rank)	Saturated fat Percent (rank)	Total sugars Percent (rank)	Fibre Percent (rank)
Beef^a^	4.3 (4)	10.0 (3)	6.5 (2)		7.8 (2)		
Bread/buns, white	8.3 (1)	6.6 (4)		12.6 (1)		3.9 (8)	16.1 (1)
Butter					6.0 (4)		
Carbonated drinks, regular	4.3 (5)			9.2 (2)		23.6 (1)	
Cereal	3.2 (10)			5.3 (5)		2.8 (10)	9.7 (2)
Cheese					6.4 (3)		
Chicken^b^	4.6 (3)	10.3 (2)	6.4 (3)		5.0 (5)		
Cold cuts/sausages	4.1 (6)	4.9 (7)	8.4 (1)		9.4 (1)		
Condiments, sweet^c^				5.7 (4)		15.7 (2)	
Eggs		5.1 (6)	5.4 (5)		4.9 (6)		
Fish		3.5 (9)					
Fried vegetables^d^	3.4 (8)		3.9 (7)	4.0 (8)	3.7 (9)		5.9 (6)
Fruit drinks				4.3 (6)		4.8 (5)	
Fruit juice						4.9 (4)	
Fruits						6.2 (3)	6.4 (4)
Game meat^e^		11.0 (1)					
Grains^f^				3.9 (9)			
Iced tea						3.9 (9)	
Margarine			5.4 (6)				
Milk						4.7 (6)	
Mixed dishes^g^		3.5 (10)			3.7 (10)		4.1 (9)
Pasta/noodles^h^	5.2 (2)	4.7 (8)		7.2 (3)			6.2 (5)
Pastries^i^				3.5 (10)		4.0 (7)	
Pizza	3.3 (9)		3.8 (9)		4.5 (7)		3.6 (10)
Pork		6.0 (5)	3.9 (8)		4.4 (8)		
Potatoes^j^				4.2 (7)			5.7 (7)
Snack foods^k^	3.7 (7)		5.6 (4)				5.1 (8)
Vegetables							9.4 (3)
Vegetable oil			3.5 (10)				

^a^Beef includes ground, steak, ribs and stewed. ^b^Chicken includes roasted, baked, fried and stewed. ^c^Condiments, sweet include sugar, jam, syrup and honey. ^d^Fried vegetables include French fries, hash browns, onion rings, and battered and deep-fried zucchini. ^e^Game meat includes moose, caribou, deer, elk, rabbit, bear, beaver, groundhog, muskrat, porcupine, goose, duck, ptarmigan, grouse and pheasant. ^f^Grains include rice, barley, quinoa and couscous. ^g^Mixed dishes include stews, pot pies, chili, ethnic and Mexican foods such as tacos and enchiladas, casseroles and salads. ^h^Pasta/noodles include plain pasta, lasagna and other mixed dishes containing pasta. ^i^Pastries include cakes, pies, muffins and doughnuts (did not include cookies). ^j^Potatoes include boiled, baked and mashed (not fried).^k^Snack foods include popcorn, chips, snack mixes (cereals, nuts), fruit leather and beef jerky

**Table 5:** Top contributors to micronutrients by percent of total and ranking for Canadian First Nations participants from the FNFNES, 2008−2018

Food	Vitamin A Percent (rank)	Vitamin C Percent (rank)	Vitamin D Percent (rank)	Folate Percent (rank)	Calcium Percent (rank)	Iron Percent (rank)	Sodium Percent (rank)	Zinc Percent (rank)
Bannock				4.5 (6)	4.9 (5)	3.0 (10)		
Beef^a^			1.9 (9)			5.8 (4)		16.3 (1)
Bread/buns, white				20.4 (1)	13.2 (2)	13.2 (1)	11.1 (2)	5.1 (3)
Butter	4.1 (6)							
Cereal				3.4 (7)		10.8 (2)		4.2 (7)
Cheese	3.9 (7)				8.9 (3)			
Chicken^b^			2.1 (8)				3.2 (9)	4.7 (4)
Cold cuts			4.3 (5)				8.8 (3)	4.3 (6)
Condiments^c^							7.1 (4)	
Cream	2.6 (9)							
Eggs	15.0 (2)		13.5 (4)	5.2 (4)	3.0 (9)	3.2 (8)		3.5 (10)
Fish			25.1 (1)					
Fried vegetables^d^		3.0 (7)						
Fruit drinks		34.3 (1)			3.5 (7)			
Fruit juice		18.5 (2)		2.5 (10)				
Fruits		10.4 (4)						
Game meat^e^	3.1 (8)	1.2 (10)				10.2 (3)		14.7 (2)
Margarine	9.4 (3)		17.1 (2)					
Milk	8.6 (4)		16.9 (3)		13.7 (1)			
Mixed dishes^f^	2.6 (10)	1.8 (9)			2.8 (10)	3.9 (6)	4.7 (5)	4.0 (8)
Pasta/noodles^g^			3.8 (6)	16.9 (2)	4.1 (6)	5.5 (5)	4.0 (7)	3.9 (9)
Pastries^h^								
Pizza				4.8 (5)	6.0 (4)	3.2 (9)	4.4 (6)	
Pork			3.5 (7)					4.4 (5)
Potatoes		5.4 (5)	1.2 (10)					
Sandwiches							3.1 (10)	
Snack foods		3.1 (6)					3.3 (8)	
Soup	5.2 (5)	2.2 (8)		2.9 (8)		3.9 (7)	12.2 (1)	
Tea				2.8 (9)				
Vegetables	22.8 (1)	11.6 (3)		5.5 (3)	3.1 (8)			

^a^Beef includes ground, steak, ribs and stewed. ^b^Chicken includes roasted, baked, fried and stewed. ^c^Condiments include sauces, ketchup, mustard, salt and vinegar. ^d^Fried vegetables include French fries, hash browns, onion rings, and battered and deep-fried zucchini. ^e^Game meat includes moose, caribou, deer, elk, rabbit, bear, beaver, groundhog, muskrat, porcupine, goose, duck, ptarmigan, grouse and pheasant. ^f^Mixed dishes include stews, pot pies, chili, ethnic and Mexican foods such as tacos and enchiladas, casseroles and salads. ^g^Pasta/noodles include plain pasta, lasagna and other mixed dishes containing pasta. ^h^Pastries include cakes, pies, muffins, and doughnuts (did not include cookies)

Twenty-four percent of adults reported taking a supplement: higher usage was reported among adults in BC (33%) and Ontario (34%). Commonly reported supplements were multivitamins/minerals and vitamin D.

## Discussion

In comparison with non-Indigenous Canadians, mean energy intakes for First Nations women were slightly higher (1864 vs. 1655–1630 kcal/day (age 19–50 years), 1669 vs. 1578 kcal/day (51–70 years) and 1664 vs. 1416 kcal/day (71 years and over)) while those for men were slightly lower (2298 vs. 2427–2236 kcal/day (age 19–50 years), 1948 vs. 2081 kcal/day (51–70 years) and 1761 vs. 1795 kcal/day (71 years and over)) (Statistics Canada 2015c). Energy intakes did seem low considering that 82% of participants were considered overweight or obese (Willett 2012); however, our estimates did not include alcohol which might account for some of the difference. The percentage of energy from protein (18.1–22.4% vs. 15.8–17.9%), fat (34.6–38.3% vs. 31.1–32.9%) and carbohydrate (47.9–52.8% vs. 46.2–50.8%) in First Nations adults compared with the population of Canada appeared higher but were mostly within the AMDR (Institute of Medicine 2005a; Statistics Canada 2015a, b). Percent energy from saturated fat was greater than the last published Canadian values for adults (Health Canada 2012).

Evaluating adequacy for nutrients with an AI is challenging so even though fibre, potassium, and linoleic acid were below the AI, the prevalence of inadequacy cannot be determined; however, these levels suggest that adults were not meeting recommendations (Institute of Medicine 2005a, 2005b). Women and men aged 19–70 years had mean intakes greater than the AI for linolenic acid, suggesting adequate intake (Institute of Medicine 2005a). It appeared that fewer First Nations adults took nutritional supplements (24%) as compared with the population of Canada where 47% of adults take them (Statistics Canada 2015d).

High fat, saturated fat, and sodium intakes in the First Nations participants in our study emphasized the need to address the quality of the diet in combatting nutrition-related chronic disease. It is critically important that we identify culturally appropriate ways to improve diet in First Nations individuals to mitigate the health risks from a high burden of NRCD such as obesity, type 2 diabetes, and cardiovascular disease (Willows et al. 2012). Many factors contribute to these risks, one of which is the nutrition transition away from TF and towards an ever-increasing share of MF and particularly UPP which are readily available and often cheaper than other alternatives (Batal et al. 2018a, b). This nutrition transition could be addressed by removing barriers and promoting the intake of TF and its role in food sovereignty and cultural connectedness (Batal et al. 2021b). First Nations individuals feel strongly about the benefits of TF (Laberge Gaudin et al. 2015; Willows 2005; Willows et al. 2018) and, as we have also demonstrated, increased intakes of these nutrient-dense foods improve the nutritional profile of First Nations individuals (Johnson-Down and Egeland 2013; Kuhnlein 1995).

Both on-reserve and off-reserve, First Nations Peoples face alarmingly high rates of food insecurity (Domingo et al. 2020; Steinhouse 2017; Willows et al. 2009, 2011). This also contributes to the burden of the nutrition transition as it points to the difficulty of obtaining nutrient-dense high-quality MF as well as TF. Incomes are lower in First Nations communities and the remoteness of some communities affects food availability and the People’s ability to purchase it due to the high cost (Batal et al. 2021a; Expert Panel on the State of Knowledge of Food Security in Northern Canada 2014; Statistics Canada 2016).

Historically, the diets of First Nations in Canada are adequate, and we know that the nutrition transition has led to a decreased intake of TF (Willows 2005). It has long been established that TF are an excellent source of nutrients, but they are increasingly difficult to obtain or are not available (Willows 2005; Willows et al. 2018, 2019). Factors such as colonial policies, climate change, industrial development, environmental contaminants and other threats to Indigenous lands have contributed to the decreased intake of these important foods (Ford 2012; Laberge Gaudin et al. 2015; Willows et al. 2019); the FNFNES has identified barriers such as the lack of a hunter, lack of resources (i.e., money or equipment/transportation), natural resource activities (e.g., mining, hydro-electricity, forestry and farming) and lack of time (Batal et al. 2021b). Overcoming these challenges is essential as even a moderate increase in TF increases the nutrient intake of First Nations (Johnson-Down and Egeland 2013). Last, the cultural and physical benefits of TF cannot be denied (Willows 2005). Nutrient contributions from TF in our study confirmed that consuming TF improved the nutrient profile of First Nations individuals. It is therefore imperative that First Nations communities explore ways to overcome the barriers to gathering and eating TF.

This was the first cross-Canada study of First Nations adults below the 60^th^ parallel and is therefore the most representative study of this population to date. Using reported diet from 24-h recalls is a strength of our study because it is an open-ended methodology (Food and Agriculture Organization 2015) and standardized methods help overcome the possible underestimation of food consumption (Livingstone and Black 2003). Despite this, it is difficult to control for underestimation or overestimation of some foods as a result of social desirability (i.e., perceived benefits of TF) or anthropometry (reported lower intakes with obesity) (Skinner et al. 2013). The recalls were conducted in the fall which may have influenced the type and amount of TF as the hunting and gathering of these are seasonal. Our data were collected over an 8-year period and may have been influenced by trends over time in food intakes occurring nationally; notably, we saw an increase in ready-to-eat foods such as UPP (Garriguet 2019; Moubarac et al. 2014). Last, our nutrient analysis software, CANDAT, does not permit us to disaggregate mixed foods such as stews and salads, influencing our analyses of nutrients from food.

## Conclusion

Many challenges stemming from the history of colonization and trauma are widespread in First Nations Peoples. First Nations Peoples in Canada face many health challenges that are nutrition-related (i.e., NRCD). It is therefore important to evaluate their diet with an aim to find culturally appropriate ways to improve health (Batal et al. 2021c). One such project took place with the Nuxalk Nation in British Columbia in the early 1980s where they were able to improve the traditional food intake and nutritional profile (Turner et al. 2013).

Despite the challenges (i.e., financial constraints, food insecurity, reduced access to healthy MF, reduced access to TF, and the contaminant burden of TF) (Willows et al. 2019), it is imperative that First Nations communities be empowered to collaboratively partner with their health practitioners to develop realistic goals for improving food sovereignty. To this end, we propose that future methods look at dietary patterns as a means to finding realistic and culturally appropriate recommendations to better the quality and nutritional value of First Nations food intake by improving TF access and use on the one hand and providing better quality store-bought foods on the other (Willows et al. 2019). To their benefit, some communities have already instituted programs to support and enhance the sustainable harvesting of TF (Cree Hunters and Trappers Income Security Board 2017; Syilx Okanagan Nation Alliance 2017).
